# The polycyclic aromatic hydrocarbon concentrations in soils in the Region of Valasske Mezirici, the Czech Republic

**DOI:** 10.1186/1467-4866-10-12

**Published:** 2009-12-14

**Authors:** Daniela Plachá, Helena Raclavská, Dalibor Matýsek, Mark H Rümmeli

**Affiliations:** 1 Centre of Nanotechnology, VSB - Technical University of Ostrava, 17.listopadu 15, 708 33 Ostrava - Poruba, Czech Republic; 2 Institute of Geological Engineering, VSB - Technical University of Ostrava, the Faculty of Mining and Geology, 17.listopadu 15, 708 33 Ostrava - Poruba, Czech Republic; 3 Leibniz Institute for Solid State and Materials Research Dresden, IFW Dresden, P.O. Box 27016, Helmholzstrase 20, 01069 Dresden, Germany

## Abstract

The polycyclic aromatic hydrocarbon (PAH) contamination of urban, agricultural and forest soil samples was investigated from samples obtained in the surroundings of Valasske Mezirici. Valasske Mezirici is a town located in the north-east mountainous part of the Czech Republic, where a coal tar refinery is situated. 16 PAHs listed in the US EPA were investigated. Organic oxidizable carbon was also observed in the forest soils. The PAH concentrations ranged from 0.86-10.84 (with one anomalous value of 35.14) and 7.66-79.39 mg/kg dm in the urban/agricultural and forest soils, respectively. While the PAH levels in the urban/agricultural soils are within the range typically found in industrialized areas, the forest soils showed elevated PAH concentrations compared to other forest soils in Western and Northern Europe. The PAH concentrations and their molecular distribution ratios were studied as functions of the sample location and the meteorological history. The soils from localities at higher altitudes above sea level have the highest PAH concentrations, and the PAH concentrations decrease with increasing distance from the town.

## 1. Background

Many sites in the Czech Republic have been negatively affected by industrial pollution. The town of Valasske Mezirici and its surroundings, in the northeast of the republic, is one of these sites. In the 1960s, a coal tar refinery was established there. Coal tar refineries are known sources of pollution; among the pollutants they produce are various organic compounds, including polycyclic aromatic hydrocarbons (PAHs).

The PAHs form a group of chemical compounds that are ubiquitous in the environment [1]. These compounds are well known for their characteristic properties, such as toxicity and carcinogenity, environmental persistence and tendency for bioaccumulation [1-5]. They form through the incomplete combustion or pyrolysis of organic matter, and their release into the atmosphere is connected with energy and heat production, local heating facilities, vehicle exhausts, refuse burning, coke ovens, and so on. They also enter the environment via natural processes such as volcanic activity or forest and prairie fires [6,7]. After being emitted into the atmosphere, they are redistributed between the gas and particle phases, and subsequently deposited to the terrestrial environment through dry or wet deposition [8]. Before being deposited, they can be transported over long distances.

Thus, the soil is contaminated with PAHs mainly from atmospheric depositions, directly or via vegetation, or in the case of arable soil with biowaste used as fertilizers [2]. Soil contamination by PAHs is considered to be a good indicator of the level of environmental pollution by human activities [8,9]. It can provide information on regional pollution sources, the long-range transport of PAHs, the rates of pollutant retention and their ultimate destination [10,11].

The aim of this paper is to investigate the occurrence of PAHs in urban and agricultural soils that are directly impacted by human activity (urban/agricultural soils) and in forest topsoils in the Valasske Mezirici Region in the Czech Republic, in the vicinity of a coal tar refinery and carbon black production. This information on PAH distribution can be used for health risk assessment and future urban development.

## 2. Materials and methods

### 2.1. Site location and description

**Figure 1:**
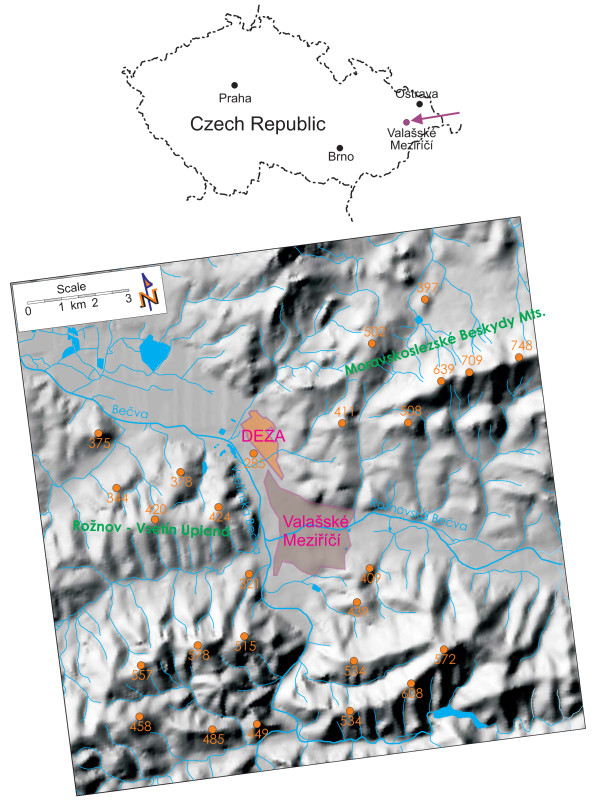
**The local landscape around the town of Valasske Mezirici town and the coal tar refinery**. Some altitudes are marked (in orange).

**Figure 2:**
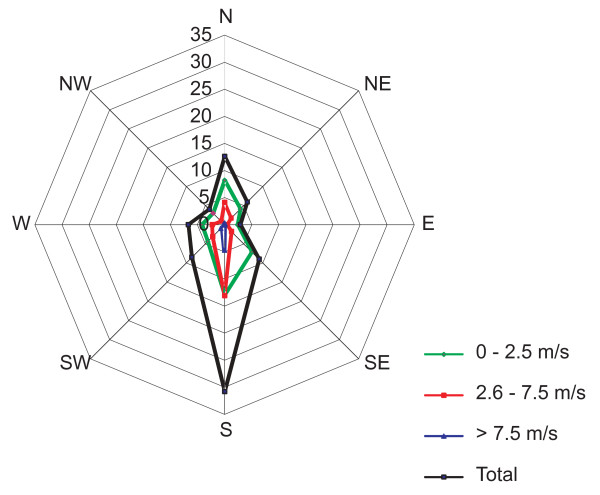
**The ten-year wind rose in the Valasske Mezirici Region**.

The study area is located in the northeast of the Czech Republic, in the district of Zlin (Figure 1). The town of Valasske Mezirici is situated at the foothills of the Moravian-Silesian Beskydy Mountains, the Hostyn Hills and the Vsetin Hills, near the confluence of the Roznov Becva and the Vsetin Becva Rivers (49°27' - 49°30'N and - 18°0' E), at altitudes 400-17°56' 600 m above sea level. The surface area is 5,481 ha [12]. According to the general ten-year wind data (Figure 2), the prevailing wind direction is from the south (30.8%), followed by calm (17.2%) and northerly wind direction (12.60%). The other wind directions account for up to 10% of the data. The eastern wind direction has the lowest frequency.

The town is an important industrial center and a significant railway and road junction, with a relatively high traffic density. The main industrial source of PAH pollution is the coal tar refinery (DEZA Corporation) and the carbon black production plant (CABOT CS). Both of these sources are located in the north of the town (Figure 1). DEZA a.s. is the only Czech producer of aromatic hydrocarbons that are obtained by processing coal tar and benzol. The annual processing capacity of DEZA is 160,000 Mt of crude benzol and 450,000 Mt of crude coal tar. The company's business is processing coal tar, mixed tar oils, benzol and raw materials for the production of phenols. Coal tars are composed of hundreds of organic compounds: aromatic compounds, including PAHs, heterocyclic PAHs, phenols, benzene, toluene, and xylenes, as well as aliphatic and polar hydrocarbons, but the chemical composition of coal tar may vary as a function of many factors, including feed stock, plant operating conditions and weathering once released to the environment [13,14].

The products of DEZA are mainly aromatic hydrocarbons - benzene, toluene, mixtures of xylenes, phenols, cresols, and xylenols - followed by polycyclic aromatic hydrocarbons - anthracene, carbazol and naphthalene, phthalic anhydride, dioctyl phthalate. Coal tar pitch is also among their important products. The company is the supplier of raw materials for the production of carbon black in the affiliated company CS-CABOT [15]. According to the Czech Integrated pollution register, the company reported annual naphthalene emissions of 50,000 kg. The emitted quantities of the other PAH compounds are not reported [16].

### 2.2 Sampling of soils and analytical methods

Forty samples were collected for the determination of the PAH distribution in soils from the area of Valasske Mezirici. Twenty samples were collected from urban grass-covered and agricultural soils in the town and in its nearest surroundings. Agricultural soil refers to soil that is used for agricultural uses, for example, soil for food production, pasture land and grassland. Urban soil refers to soil not used for agriculture, occurring in the territory of the urban settlement. In this context, it represents soil in town gardens, soil along the roads and road junctions, parking places, and so on. These type of soils are affected by human activities [17,18]. The sampling sites were selected in order to reflect the diverse exposure of soils to the pollution sources and dominant wind directions. They represent samples directly affected by industrial emissions, and directly subjected to transport and/or local heating emissions.

Twenty samples were collected from the humus layer of forest soils on the hill slopes in the surroundings of the town; 15 in coniferous forests and 5 in mixed forests. The sampling sites were also selected to represent a diversity of dominant wind directions.

**Figure 3:**
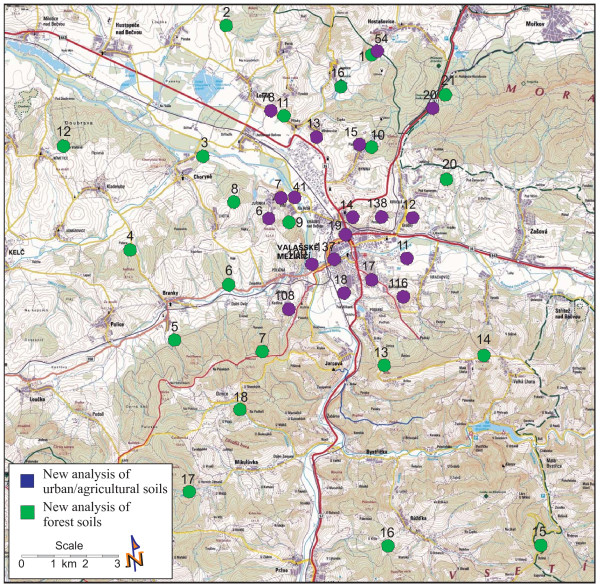
**Locations of sampling sites in the Valasske Mezirici Region**.

The sampling sites are shown in Figure 3 and described in Tables 1 and 2. The sampling procedure, the physical sample preparations and the representative sample division were accomplished by using simple random shallow sampling according to Tan (2005) and ISO 10381-4:2003 and ISO 10381-5:2005 [19-21]. The samples were collected from the surface layer (from A-horizon of 0-10 cm depth), after removing grass in urban/agricultural soil and needles and litter in forest soil. Approximately 2 kg of material were collected from a square area of 10 × 10 m in each sampling site. All samples were put into glass containers with a minimum headspace of air, and immediately transported to the laboratory. In each series of samples, one sample was collected to the depth of the A- and B- (14-50 cm) horizons.

**Table 1:** Description of urban/agricultural soil sampling sites

Sample ID	Soil sample type	Altitude (m a.s.l.)	Coordinates X.Y		Direction*	pH/KCl	pH/H_2_0
6	urban	441	499076	1138446	South-West	6,61	5,79
7	agricultural	427	498619	1137867	South	7,45	7,03
11	urban	181	495031	11440205	South-East	6,62	5,83
12	urban	409	494700	1138994	South-East	6,19	4,95
13	agricultural	192	497293	1136156	North	6,14	5,08
14	urban	403	496516	1138738	South	7,24	6,98
15	agricultural	350	496016	1136550	East	6,28	4,74
16	agricultural	352	496356	1134721	North	7,04	6,36
17	urban	316	496189	1140722	South	6,63	5,95
18	urban	318	497084	1141013	South	6,48	5,93
19	urban	336	496803	1139240	South	5,71	4,69
20	semi-urban	400	493484	1135740	North-East	6,10	5,06
41	urban	207	498198	1137905	South	6,73	5,83
54	agricultural	337	494944	1133779	North-East	6,80	5,76
78	agricultural	313	498421	1135172	North	5,55	4,62
108	agricultural	359	498827	1141290	South	6,13	5,28
109	urban	300	497943	1140006	South	7,68	7,12
116	agricultural	357	495296	1141360	South-East	6,60	5,68
137	urban	321	497250	119944	South	7,30	6,86
138	urban	404	495636	11338848	East	6,86	5,92

*direction from coal tar refinery

**Table 2:** Description of forest soil sampling sites

Sample ID	Altitude (m a.s.l.)	Coordinates X.Y		Direction*	Distance**	pH/KCl	pH/H_2_0	Organic ox carbon (%)
1	400	495130.11	1133851.02	Nord	5,6	2,85	3,30	15,60
2	290	499432.48	1132383.39	Nord	7,0	2,88	3,35	18,01
3	275	500649.60	1136282.43	West	5,2	2,88	3,41	13.89
4	350	503261.27	1138848.46	West	6,5	3,06	3,51	11,05
5	400	502271.46	1141773.46	South-West	6,1	3,16	3,66	19,20
6	330	500403.36	1140301.10	South-West	3,8	3,06	3,59	10,25
7	500	499647.69	1142466.15	South	4,1	2,90	3,80	12,90
8	365	499887.02	1137853.47	West	3,6	3,35	3,84	10,31
9	400	498311.52	1138633.84	South	2,1	3,06	3,52	8,88
10	300	495502.65	1136669.43	East	3,1	2,81	3,24	16.45
11	295	498040.78	1135367.05	Nord-West	4,7	3,47	4,03	10,59
12	350	504876.07	1135413.47	West	9,3	3,23	3,71	10,31
13	450	495988.96	1143370.04	South	4,1	3,23	3,61	3,45
14	625	492916.90	1143460.66	South-East	5,7	3,10	3,48	13.54
15	670	491938.90	1149477.01	South	11,4	2,82	3,31	17.21
16	550	496606.90	1148941.20	South	9,7	3,04	3,49	15.78
17	400	502434.54	1146456.76	South	9,1	2,99	3,50	14.27
18	400	500554.75	1144153.94	South	6,4	2,74	3,26	18.91
20	400	493353.48	1137947.81	East	3,6	3,12	3,52	16.47
21	400	493050.59	1135369.90	Nord East	5,3	3,09	3,38	17.85

* direction from coal tar refinery

* *approximate distance of the middle town.

Analyses of 16 PAHs identified by the US Environmental Protection Agency (EPA) as priority pollutants were performed at the Institute of Public Health, The Department of Hygienic Laboratories in Frydek-Mistek, working in accordance with EN ISO/IEC 17025 and accredited by The Czech Accreditation Institute. The laboratory is the Czech National Reference Laboratory for Persistent Organic Pollutants.

All the solvents used (purchased from Sigma Aldrich) were of HPLC grade. The stock reference standard mixture of 16 PAH EPA 610 PAH MIX (Supelco Co., Cat. No. 48743), including naphthalene, acenaphthylene, acenaphthene, fluorene, phenanthrene, anthracene, fluoranthene, pyrene, benz [a]anthracene, chrysene, benzo [b]fluoranthene, benzo [k]fluoranthene, benzo [a]pyrene, indeno [1,2,3-cd]pyrene, dibenzo [a,h]anthracene and benzo [ghi]perylene of 100-2000 ng/ml in methanol, was used as an external calibration standard. The calibration was performed by direct injection of diluted standard solutions into GC or HPLC devices, and the regressions of peak areas against concentrations were calculated (R^2 ^> 0.9998). The standards were simultaneously treated using all procedure steps to eliminate losses of PAH compounds during sample preparations and extractions. No surrogate standard was used, but matrix influence was followed for different PAH-spiked materials (sewage sludge, different soils, ashes, rubbish).

The limits of detection and measurement uncertainties for PAH compounds are shown in Table 3. The values of the limits of detection were derived 1) from the smallest integrable areas for each compound peaks in signal and 2) from the ratio between the analyte signal and the baseline noise (limit of detection = peak height/noise ratio of 3). The limits of detection were determined for contaminated soil matrices [22]. The measurement uncertainties are expanded uncertainties calculated by multiplying the combined standard uncertainties by a coverage factor k = 2 for an approximate level of confidence of 95% [23].

**Table 3:** Limit of detection and measurement uncertainty

PAHs	Limit of detection mg/kg	Measurement uncertainty* %
Naphthalene	0.050	30
Acenaphthylene	0.050	30
Acenaphthene	0.050	30
Fluorene	0.050	30
Phenanthrene	0.010	20
Anthracene	0.005	20
Fluoranthene	0.005	20
Pyrene	0.005	20
Benzo [a]anthracene	0.005	20
Chrysene	0.005	20
Benzo [b] fluoranthene	0.005	20
Benzo [k] fluoranthene	0.005	20
Benzo [a] pyrene	0.002	20
Dibenzo [a.h] anthracene	0.010	20
Benzo [ghi] perylene	0.005	20
Indeno [123-cd] pyrene	0.010	20

* The measurement uncertainties are expanded uncertainties calculated by multiplying the combined standard uncertainties by a coverage factor k = 2 for an approximate level of confidence of 95%.

A sufficient part of each sample was taken for the PAH extraction. The samples were not dried before extraction. The PAH compounds were extracted from the soil samples using a 1:1 (v:v) mixture of acetone and hexane in a Soxwav 3.6 microwave extractor (5 min of 20% microwave performance, following 55 min of 60% performance). The final extracts were filtered, dried with Na_2_SO_4 _and evaporated to produce near-dry residue. The residue was redissolved in 1 ml of hexane and divided into two aliquots; one half of the volume was used for gas chromatography - mass spectrometry (GC/MS) analysis and the second half was analyzed by high pressure liquid chromatography with fluorescence detector (HPLC/FD) after solvent exchange (hexane for methanol). The chromatographic analyses were performed using a GCQ/Polaris Q ThermoFinnigan gas chromatograph, equipped with a DB-5MS 30 m × 0.25 mm × 0.25 μm column, and a Hewlett Packard Series 1100 liquid chromatograph with a fluorescence detector, equipped with a Merck LiChroCART 250-3 column. The GC/MS analysis was used for the determination of light PAHs - naphthalene, acenaphthene, acenaphthylene and fluorene; the other PAHs were analyzed by HPLC/FD method.

The dry matter content of each soil sample was determined after drying in an oven at 105 ± 3°C to constant weight. The organic oxidizable carbon (C_ox_) content (in percentage) was determined in each forest soil sample by the modified Walkley-Black (VB) dichromate methods [24]. The values were determined to be in the range 3.45-19.20% (Table 2). ISO 10390:2005 was used for the routine determination of pH using a glass electrode in a 1:5 (volume fraction) suspension of soil in water (pH in H_2_O) and 1 mol.l^-1 ^potassium chloride solution (pH in KCl) (Table 1 and 2).

## 3. Results and discussion

### 3.1 PAH content in urban/agricultural soil

The results of the PAH analysis of urban/agricultural soil samples are shown in Tables 4, 5 in mg/kg dm, and the statistical evaluation appears in Table 6. The total concentrations of PAHs (viz. the selected 16 PAHs) varied from 0.861-10.840 mg/kg dm, with one anomalous value of 35.140 mg/kg dm; the arithmetic mean of the PAH concentrations was 5.527 mg/kg dm and the median was 3.370 mg/kg dm. Only the concentrations of those PAHs with values above the detection limits are calculated. The sum of 7 carcinogenic PAHs (according to IARC - benzo [a]anthracene, chrysene, benzo [b]fluoranthene, benzo [k]fluoranthene, benzo [a]pyrene, dibenzo [a,h]anthracene, indeno [1,2,3-cd]pyrene) ranged from 0.400 to 5.090 mg/kg dm, with one value of 16.000 mg/kg dm belonging to the anomalous PAH concentration. The sum of these 7 carcinogenic PAHs formed 40-57% of the total PAHs; only one sample from locality Krhova (ID sample 138) had the sum 30%. An analysis of the sample with A- and B-horizons is shown in Table 7. The observed values of the PAH concentrations in the B-horizon were similar to the concentrations in the A-horizon. The values were in the range that was observed for typical concentrations in urban soils (0.600-3.000 mg/kg), and they are higher than typical concentrations in forest and rural soils (up to 1.000 mg/kg) [25]. We do not know the full history of the site, but the relatively high PAH concentration values show an influence due to human activity. The concentrations of the carcinogenic fraction - benzo [a]pyrene were in the range of 0.06-2.25 mg/kg dm, with an arithmetic mean of 0.36 mg/kg dm and a median of 0.22 mg/kg dm.

**Table 4:** PAH concentrations in selected urban/agricultural soils

Sample ID/locality	6/Jurinka	7/Jurinka orchard	11/Valmez swimming pool	12/Hrachovec	13/Přiluky	14/Obora III	15/Bynina	16/Jasenice	17/Valmez hospital	18/Valmez South
PAHs	mg/kg dm									
Naphthalene	0.100	0.400	0.190	0.120	0.720	0.100	0.140	0.130	0.470	1.050
Acenaphthylene	0.006	< 0.005	0.006	0.003	0.007	0.007	< 0.005	< 0.005	0.007	0.015
Acenaphthene	0.074	0.062	0.048	0.025	0.050	0.058	0.020	0.190	0.036	0.073
Fluorene	0.071	0.052	0.037	0.028	0.047	0.046	0.020	0.110	0.030	0.064
Phenanthrene	0.350	0.180	0.180	0.057	0.170	0.290	0.054	0.074	0.270	0.370
Anthracene	0.047	0.030	0.041	0.009	0.036	0.046	0.009	0.012	0.066	0.044
Fluoranthene	1.000	0.350	0.830	0.160	0.390	0.610	0.120	0.210	0.830	0.800
Pyrene	0.700	0.240	0.620	0.120	0.280	0.410	0.087	0.150	0.620	0.560
Benzo [a] anthracene	0.320	0.150	0.310	0.071	0.190	0.240	0.054	0.096	0.430	0.300
Chrysene	0.460	0.230	0.440	0.100	0.280	0.330	0.079	0.140	0.600	0.450
Benzo [b] fluoranthene	0.240	0.140	0.230	0.053	0.180	0.180	0.053	0.082	0.310	0.240
Benzo [k] fluoranthene	0.150	0.082	0.140	0.032	0.110	0.110	0.029	0.048	0.190	0.140
Benzo [a] pyrene	0.290	0.160	0.280	0.060	0.220	0.210	0.056	0.093	0.400	0.260
Dibenzo [a.h] anthracene	0.220	0.140	0.240	0.052	0.180	0.160	0.050	0.080	0.300	0.210
Benzo [ghi] perylene	0.070	0.039	0.063	0.012	0.050	0.048	0.011	0.019	0.093	0.055
Indeno [123-cd] pyrene	0.440	0.260	0.440	0.083	0.320	0.310	0.079	0.130	0.550	0.390
**Σ 16 PAHs**	**4.538**	**2.515**	**4.095**	**0.985**	**3.230**	**3.155**	**0.861**	**1.564**	**5.202**	**5.021**
**Σ 7 carcinogenic PAHs**	**2.120**	**1.162**	**2.080**	**0.451**	**1.480**	**1.540**	**0.400**	**0.669**	**2.780**	**1.990**

**Table 5:** PAH concentrations in selected urban/agricultural soils

Sample ID/locality	19/Valmez castle	20/backround	41/near coal tar refinery	54/Hostašovice	78/Vysoká	108/Poličná	109/Valmez confluence	116/on Štěpánov	137/Valmez Janáčkova	138/Krhová
PAHs	mg/kg dm									
Naphthalene	0.230	0.230	0.180	0.220	0.200	0.180	0.210	0.210	0.440	0.500
Acenaphthylene	0.007	0.013	0.007	0.007	0.006	0.006	< 0.05	< 0.05	< 0.04	0.022
Acenaphthene	0.065	0.230	0.060	0.039	0.027	0.065	0.150	0.250	0.680	0.084
Fluorene	0.049	0.150	0.043	0.035	0.032	0.050	0.083	0.210	0.480	0.050
Phenanthrene	0.210	0.140	0.280	0.220	0.200	0.550	0.490	3.300	0.760	0.078
Anthracene	0.027	0.015	0.045	0.030	0.025	0.120	0.160	0.720	0.140	0.008
Fluoranthene	0.470	0.440	0.640	0.660	0.500	1.200	2.000	8.300	1.800	0.140
Pyrene	0.330	0.310	0.460	0.530	0.360	0.800	1.400	5.700	1.300	0.100
Benzo [a] anthracene	0.180	0.170	0.320	0.250	0.200	0.490	0.750	2.600	0.780	0.057
Chrysene	0.310	0.280	0.480	0.380	0.310	0.740	1.000	3.500	1.100	0.093
Benzo [b] fluoranthene	0.210	0.170	0.300	0.210	0.180	0.390	0.470	1.600	0.570	0.058
Benzo [k] fluoranthene	0.110	0.097	0.170	0.120	0.110	0.240	0.300	1.100	0.350	0.031
Benzo [a] pyrene	0.190	0.180	0.340	0.220	0.220	0.460	0.610	2.300	0.720	0.056
Dibenzo [a.h] anthracene	0.180	0.160	0.280	0.190	0.200	0.370	0.470	1.800	0.580	0.050
Benzo [ghi] perylene	0.041	0.039	0.081	0.049	0.047	0.096	0.130	0.450	0.150	0.012
Indeno [123-cd] pyrene	0.320	0.270	0.530	0.350	0.340	0.630	0.860	3.100	0.990	0.077
**Σ 16 PAHs**	**2.929**	**2.894**	**4.216**	**3.510**	**2.957**	**6.387**	**9.083**	**35.140**	**10.840**	**1.416**
**Σ 7 carcinogenic PAHs**	**1.500**	**1.327**	**2.420**	**1.720**	**1.560**	**3.320**	**4.460**	**16.000**	**5.090**	**0.422**

**Table 6:** Statistical evaluation of PAH concentrations in urban/agricultural soils

PAHs	Unit	Minimum	Maximum	Arithmetic mean	Standard deviation	Geometric mean	Median	Number of samples
Naphthalene	mg/kg dm	0.100	1.050	0.301	0.233	0.241	0.210	20
Acenaphthylene	mg/kg dm	0.003	0.022	0.009	0.005	0.008	0.007	20
Acenaphthene	mg/kg dm	0.020	0.680	0.114	0.145	0.073	0.064	20
Fluorene	mg/kg dm	0.020	0.480	0.084	0.101	0.059	0.050	20
Phenanthrene	mg/kg dm	0.054	3.300	0.411	0.685	0.234	0.215	20
Anthracene	mg/kg dm	0.008	0.720	0.082	0.152	0.039	0.039	20
Fluoranthene	mg/kg dm	0.120	8.300	1.073	1.730	0.603	0.625	20
Pyrene	mg/kg dm	0.087	5.700	0.754	1.187	0.431	0.435	20
Benzo [a] anthracene	mg/kg dm	0.054	2.600	0.398	0.542	0.246	0.245	20
Chrysene	mg/kg dm	0.079	3.500	0.565	0.725	0.364	0.355	20
Benzo [b] fluoranthene	mg/kg dm	0.053	1.600	0.293	0.327	0.205	0.210	20
Benzo [k] fluoranthene	mg/kg dm	0.029	1.100	0.183	0.226	0.122	0.115	20
Benzo [a] pyrene	mg/kg dm	0.056	2.300	0.366	0.475	0.236	0.220	20
Dibenzo [a.h] anthracene	mg/kg dm	0.050	1.800	0.296	0.370	0.197	0.195	20
Benzo [ghi] perylene	mg/kg dm	0.011	0.450	0.078	0.093	0.051	0.050	20
Indeno[123-cd)pyrene	mg/kg dm	0.077	3.100	0.523	0.636	0.347	0.345	20
**Σ 16 PAHs**	mg/kg dm	0.861	35.140	5.527	7.221	3.665	3.370	20
**Σ 7 carcinogenic PAHs**	mg/kg dm	0.400	16.000	2.625	3.299	1.720	1.640	20

**Table 7:** PAH concentration in A- and B- horizons of urban/agricultural and forest soil in the Valasske Mezirici Region

Sample ID/locality	Štěpánov (19) Agricultural soil A-horizon	Štěpánov (19) Agricultural soil B-horizon	Hodslavice (21) Forest soil A-horizon	Hodslavice (21)/Forest soil B-horizon
PAHs	mg/kg dm			
Naphthalene	0.008	0.009	0.32	0.006
Acenaphthylene	0.004	0.007	< 0.10	0.003
Acenaphthene	0.009	< 0.008	0.25	0.011
Fluorene	< 0.020	< 0.016	0.12	< 0.020
Phenanthrene	0.11	0.093	1.3	0.02
Anthracene	0.015	0.009	0.13	0.002
Fluoranthene	0.26	0.25	3.2	0.044
Pyrene	0.19	0.18	2	0.031
Benzo [a] anthracene	0.099	0.088	0.97	0.013
Chrysene	0.17	0.15	2.2	0.034
Benzo [b] fluoranthene	0.13	0.092	1.8	0.037
Benzo [k] fluoranthene	0.061	0.049	0.78	0.012
Benzo [a] pyrene	0.12	0.093	1.1	0.015
Dibenzo [a.h] anthracene	0.085	0.055	1.1	0.016
Benzo [ghi] perylene	0.018	0.012	0.26	0.004
Indeno [123-cd] pyrene	0.17	0.11	1.9	0.029
**Σ 16 PAHs**	1.469	1.221	17.53	0.297
**Σ 7 carcinogenic PAHs**	0.835	0.637	9.850	0.156
c_ox _(%)	3.82	1.78	17.85	4.58

c_ox _content of organic oxidable carbon in soil samples (%)

The highest concentrations of the 16 selected PAHs were identified in the urban soils in the Valasske Mezirici town territory (ID 137, 109, 17, 18, 41, 11), and at the Jurinka (ID 6) and Policna (ID 108) sites. All of these sampling sites are situated south of the coal tar refinery and are in the proximity of the town center and the coal tar refinery. Influences from all PAH sources (industrial activity, local heating and transport) are evident. The remaining samples were collected north of the study area, and the identified PAH concentrations were lower in these sites. The sites (ID 13, 15, 20, 54, 78) are situated in the direction of the prevailing wind flow, and the landscape of this region decreases in altitude in this direction. The PAH atmospheric dispersion is greater in this direction, and a lower level of PAH deposition occurs at these sites.

**Figure 4:**
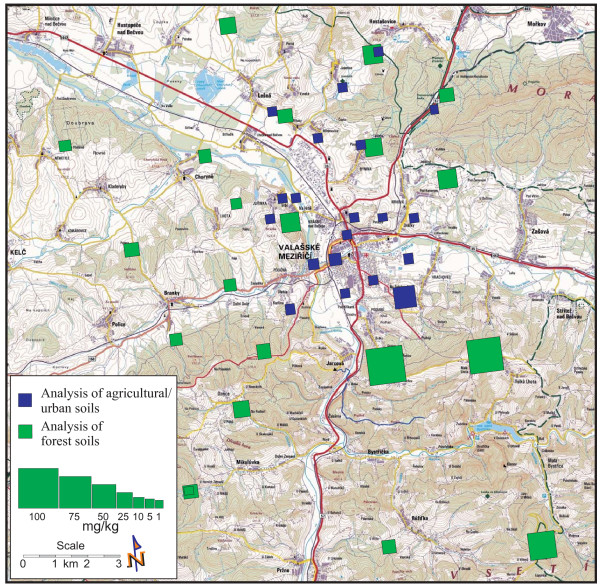
**The PAH concentrations in urban/agricultural and forest soils in the Valasske Mezirici Region**.

**Figure 5:**
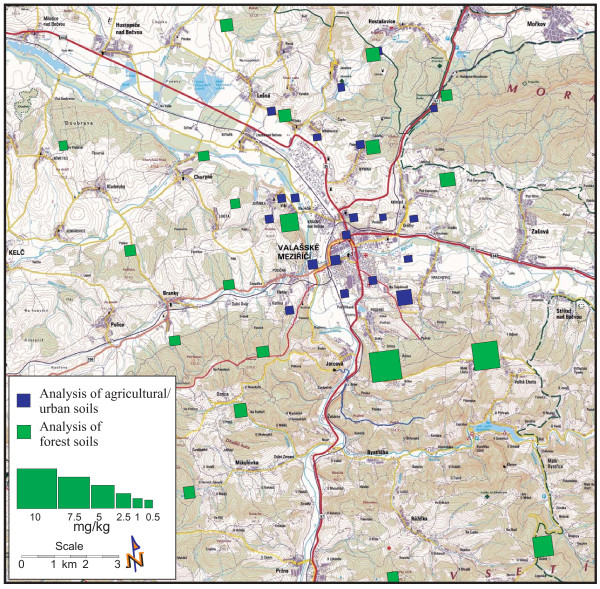
**The benzo [a]pyrene concentrations in urban/agricultural and forest soils in the Valasske Mezirici Region**.

Anomalous concentrations of the PAHs (35.16 mg/kg dm) and benzo [a]pyrene (2.25 mg/kg dm) were identified in the sample ID 116, in Stepanov. The values of the PAHs were significantly higher than in the other soil samples. This sample was collected in agricultural soil on the slope above the town in the southeast direction. It suggests that PAH deposition at higher altitudes in the proximity of PAH sources can occur as a consequence of unfavorable meteorological conditions associated with the low wind directions or calm conditions that are frequent in this region (see 2.1). These conditions are favorable for the occurrence of near ground high pollutant concentrations and are not appropriate for good atmospheric dispersion of pollutants [26]. Graphical presentations of the results are shown in Figures 4 and 5, illustrating the concentrations of the studied PAHs and benzo [a]pyrene in urban/agricultural as well as forest soils.

**Figure 6:**
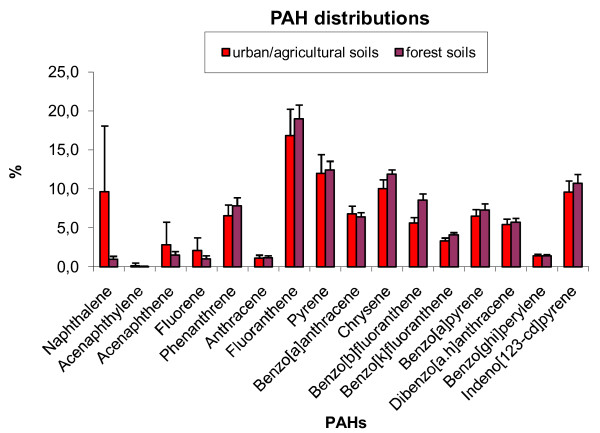
**Comparison of PAH percentage distributions in urban/agricultural and forest soils in the Valasske Mezirici Region**.

The average PAH16 distribution pattern is shown in Table 8 and in Figure 6. Fluoranthene, pyrene, chrysene and indeno [1,2,3-cd]pyrene are the most prominent compounds in the samples (calculated from average values). The predominance of 4-6 ring compounds in the soil samples suggests pyrogenic sources [27]. However, higher concentrations of naphthalene were found in 12 samples (IDs 7,12,13,15,16,17,18,19,20,54,78,138). Six of these samples were collected in the coal tar refinery surroundings (IDs 7,13,15,16,54,78), four were collected in the town territory (IDs 12,17,18,19) in the proximity of the main routes, and one (ID 20) was from the background site. The proportion of naphthalene in the PAH samples was generally in the range of 6.2-22.3%, and in one case even 35.3% (ID 138).

**Table 8:** The percentage distribution of each PAH compound in total PAH concentration

PAHs	Percentage (%)		PAHs	Percentage (%)	
	Urban/agricultural soil	Forest soil		Urban/agricultural soil	Forest soil
Naphthalene	0.60-16.20	0.38-1.95	Benzo [a] anthracene	5.81-8.30	5.29-8.06
Acenaphthylene	0.13-0.45	0.06-0.71	Chrysene	5.34-11.57	10.01-12.71
Acenaphthene	0.69-12.17	0.73-2.45	Benzo [b] fluoranthene	4.87-6.21	6.98-10.27
Fluorene	0.58-5.19	0.47-2.26	Benzo [k] fluoranthene	3.27-4.07	3.40-4.56
Phenanthrene	4.44-9.26	4.79-9.44	Benzo [a] pyrene	5.20-7.62	6.27-9.83
Anthracene	0.79-1.45	0.74-1.79	Benzo [ghi] perylene	4.11-6.67	4.72-6.67
Fluoranthene	12.30-22.14	14.40-22.65	Dibenzo [a,h] anthracene	1.10-1.59	1.11-1.83
Pyrene	8.72-16.30	9.48-13.86	Indeno [1,2,3-cd] pyrene	7.67-12.62	8.76-13.34

The presence of 2- and 3- ring PAHs can indicate more recent PAH deposition [27]. It is well known that light PAH compounds occur in soils in lower proportions than heavier ones, due to their physico-chemical properties, such as higher water solubility, volatility and biodegradability and lower sorption ability to soil organic matter or particles [28,29]. Removal of the PAHs from the atmosphere is accomplished by dry or wet deposition of particles and vapors [30,31]. Generally, it is known that for high level sources, the level of deposition near the source is small and increases with downwind distance from the source until it reaches a maximum, after which it decreases. For ground-level sources, the highest dry deposition is directly next to the source, and decreases downwind. Thus, it is possible to explain higher naphthalene contents in the soils in the territory of Valasske Mezirici due to naphthalene in PAH emissions from transport, local heating and the coal tar refinery.

Diagnostic ratios of selected PAH compounds are generally considered to be a good indicator of the pollution sources and of the mechanism of PAH transport into the soil. The ratios Ind/(Ind+BghiP) and Flt/(Flt+Pyr) are often used to distinguish between pyrogenic and petrogenic sources. The value of Ind/(Ind+BghiP) > 0.5 indicates grass/coal/wood combustion sources. The values of Flt/(Flt+Pyr) > 0.4 indicates pyrogenic sources, and we can distinguish between the values 0.4-0.5 for fuel combustion and > 0.5 for grass/coal/wood combustion sources. The ratio BaP/BghiP can also be used as an indicator for the determination of traffic and non-traffic sources, when the value > 0.6 is characteristic of traffic sources [2,11]. The values of Ant/(Ant+Phe) < 0.1 and Baa/(Baa+Chry) < 0.2 correspond to petrogenic sources; values > 0.1 and >0.35, respectively, indicate pyrogenic sources. The values of the ratio Baa/(Baa+Chry) between 0.20-0.35 indicate mixed petrogenic and pyrogenic sources [32,33].

**Figure 7:**
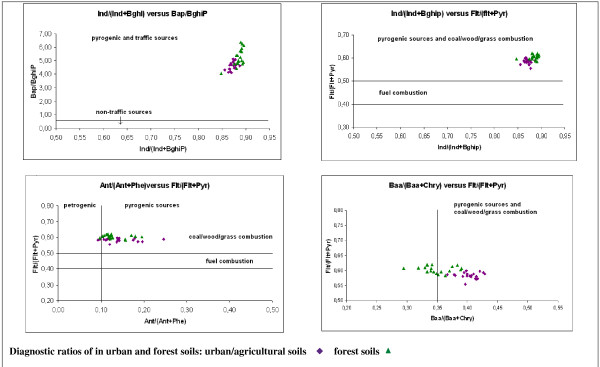
**The comparison of molecular diagnostic ratios**.

In our data set, the values of Ind/(Ind+BghiP) range between 0.86-0.89, those of Flt/(Flt+Pyr) are in the range of 0.55-0.60 and those of BaP/BghiP are between 4.10-5.11. The values of Ind/(Ind+BghiP) and BaP/BghiP are significantly higher than those presented in Maliszewska-Kordybach et al. (2008), where the values are in the ranges of 0.45-0.7, 0.3-0.6 and 0.7-1.4, respectively [2]. The characteristic ratios indicate predominantly pyrogenic sources and coal/wood/grass combustion. The values of Ant/(Ant+Phe) are in the range of 0.09-0.2, and the Baa/(Baa+Chry) ratio ranges from 0.37-0.43. These ratio values confirmed that pyrogenic sources are the main pollution sources, even in the case of ID 116, where the highest PAH contents were determined. All these diagnostic ratios are illustrated in Figure 7 and in Table 9.

**Table 9:** Characteristic PAH molecular diagnostic ratios for urban/agricultural and forest soils in Valasske Mezirici Region compared with [50]

Molecular diagnostic ratio values				
Diagnostic ratio	Urban/agricultural	Forest soil	**Coal Tar **[16]	Sources
Ant/(Ant + Phe)	0.09-0.25	0.10-0.20	0.22-0.27	pyrogenic (1 sample petrogenic)
Baa/(Baa + Chry)	0.37-0.43	0.29-0.39	0.48-0.58	pyrogenic > 0. 35. petrogenic < 0.2.;10 samples of forest soils > 0.2 and < 0.35
Flt/(Flt + Pyr)	0.55-0.60	0.59-0.62	0.36-0.58	pyrogenic and coal/wood/grass combustion
Ind(Ind + BghiP)	0.86-0.89	0.85-0.90	0.38-0.59	pyrogenic and coal/wood/grass combustion
BaP/BghiP	4.10-5.11	4.07-6.39	1.67-3.25	traffic

The percentage of the sum of 8 PAHs compounds (with 4 or more aromatic rings, excluding fluoranthene and pyrene) and the sum of naphthalene and phenanthrene concentrations from the full set of PAHs are alternative ways to determine the pollution source [34]. The percentages of the naphthalene and phenanthrene sum from the PAHs under investigation were in the range 9-28.3%, except in one case (sample ID 138), where the value was 40%. The contribution of compounds with 4 or more aromatic rings was in the range of 41-59%. These values are in accordance with the data presented in Wilcke et al. (2007a), where soil evaluations were performed in Switzerland, Germany, Spain, Korea and China, and combustion and long range transport were identified as the main pollution sources in this study.

The Czech Republic requires the regular monitoring of agricultural soils [35]. The PAHs level ranged from 0.056 to 9.489 mg/kg dm for PAHs (16 except acenaphthylene) and < 0.004-0.624 mg/kg dm for benzo [a]pyrene, with annual arithmetic means ranging from 0.797-1.297 mg/kg dm for PAHs (16 except for acenaphthylene) and from 0.059-0.119 mg/kg dm for benzo [a]pyrene. The annual median values were in the range of 0.534-0.967 mg/kg dm for PAHs and 0.047-0.089 mg/kg dm for benzo [a]pyrene. The comparison of the arithmetic mean and median values with those identified in the agricultural and urban soils in Valasske Mezirici confirm a higher load of the PAHs pollution in the Valasske Mezirici Region.

These values were compared to the data reported in Maliszewska-Kordybach et al. (2008) for the top layer of soils from non-industrial areas in Europe. Our minimum values are significantly higher than the reported data set, but the maximum values (except the sample ID 116) are comparable with those in Norway (0.009-11.000 mg/kg dm), in the UK (0.042-11.200 mg/kg dm), and in Poland (0.075-11.391 mg/kg dm) [2].

### 3.2 PAHs in forest soils

Two processes affect the fate of PAHs in forest soils: deposition and decomposition. In addition to the PAHs input to the soil through litter, dry and wet deposition of aerosol particles is an important pathway of path contamination of acid forest soils. The highest PAH concentrations in the air were in aerosol particles of grain size of 0.1-3 μm[36]. PAHs may be degraded in forest soils/forest humus, although microbial degradation activity decreases at pH levels below 3.6 [37,38]. The scavenging effect is a key source of forest soil contamination, and the high amount of natural organic matter in forest soil also provides better conditions for the binding and accumulation of PAHs in soils [8,29,39]. Coniferous trees with needles are more efficient at collecting particles and cloud droplets than leafy trees [30].

The observed PAH concentrations in forest soils are summarized in Tables 10, 11 and the statistical evaluation is in Table 12. The PAH concentrations varied from 7.657-79.385 mg/kg dm, with arithmetic mean 25.51 mg/kg dm and median 18.21 mg/kg dm. The benzo [a]pyrene concentrations were in the range of 0.53-6.20 mg/kg dm, with arithmetic mean 1.85 and median 1.30 mg/kg dm. The results of the analysis of A- and B-horizons for forest soil sample are shown in Table 7. The PAH content of the B-horizon are higher than those corresponding to typical forest soils (0.005-0.100 mg/kg), which showed a higher pollution load in the forest soils in the region [25]. All the analyzed PAHs were identified in each soil sample, except acenaphthylene, which was below the detection limit in most samples.

**Table 10:** PAH concentrations in selected forest soils

Sample ID/locality	1/Nad Lipuvkou	2/Haj	3/Choryne	4/Branky-Kladeruby	5/Branky-Studanky	6/Zavadilka	7/Policna	8/Lhota u Choryne	9/Jurinka	10/Bynina
PAHs	mg/kg dm									
Naphthalene	0.360	0.250	0.180	0.120	0.096	0.110	0.190	0.150	0.350	0.350
Acenaphthylene	<0.07	<0.08	0.038	<0.04	<0.08	<0.06	<0.09	<0.03	<0.07	<0.05
Acenaphthene	0.640	0.244	0.150	0.330	0.140	0.190	0.150	0.110	0.450	0.220
Fluorene	0.670	0.160	0.120	0.210	0.084	0.100	0.110	0.081	0.280	0.170
Phenanthrene	2.700	1.800	1.000	1.400	0.950	0.730	1.300	0.570	2.100	2.000
Anthracene	0.500	0.250	0.120	0.150	0.120	0.150	0.170	0.076	0.510	0.280
Fluoranthene	5.700	4.000	2.200	3.400	2.100	1.900	3.100	1.400	4.100	4.800
Pyrene	4.000	2.700	1.400	2.300	1.300	1.200	1.900	0.900	2.700	3.100
Benzo[a] anthracene	1.900	1.300	0.690	1.000	0.650	0.720	1.000	0.450	2.100	1.600
Chrysene	3.500	2.600	1.400	1.900	1.300	1.200	2.000	0.960	3.300	3.100
Benzo[b] fluoranthene	2.300	2.000	0.990	1.300	1.100	0.880	1.400	0.710	2.200	2.300
Benzo[k] fluoranthene	1.100	0.950	0.490	0.640	0.480	0.440	0.690	0.320	1.300	1.100
Benzo[a] pyrene	1.900	1.500	0.840	1.100	0.800	0.860	1.100	0.530	2.800	1.700
Dibenzo[a.h] anthracene	1.400	1.300	0.710	0.900	0.670	0.640	1.000	0.460	1.900	1.400
Benzo[ghi] perylene	0.330	0.310	0.170	0.230	0.160	0.140	0.230	0.110	0.520	0.340
Indeno[123-cd ] pyrene	2.600	2.300	1.300	1.600	1.300	1.200	1.900	0.830	3.800	2.600
**Σ 16 PAHs**	**29.600**	**21.664**	**11.798**	**16.580**	**11.250**	**10.460**	**16.240**	**7.657**	**28.410**	**25.060**
**Σ 7 carcinogenic PAHs**	**14.700**	**11.950**	**6.420**	**8.440**	**6.300**	**5.940**	**9.090**	**4.260**	**17.400**	**13.800**
C_ox _(%)	15.600	18.010	13.890	11.050	19.200	10.250	12.900	10.310	8.880	16.450

C_ox _content of organic oxidizable carbon in soil samples (%)

**Table 11:** PAH concentrations in selected forest soils

Sample ID/locality	11/Priluky	12/Doubrava	13/Podlesi	14/Velka Lhota	15/Dusna	16/Ruzdka	17/Mikuluvka	18/Oznice	20/Krhova-Jehlicna
PAHs	mg/kg dm								
Naphthalene	0.190	0.110	0.360	0.290	0.200	0.110	0.100	0.120	0.160
Acenaphthylene	<0.03	<0.04	0.055	0.043	0.043	<0.07	0.023	0.049	0.032
Acenaphthene	0.300	0.190	0.580	1.200	1.300	0.190	0.310	0.180	0.360
Fluorene	0.210	0.088	0.370	1.000	1.000	0.086	0.170	0.170	0.220
Phenanthrene	1.400	0.660	3.800	6.900	5.000	0.950	1.100	1.700	2.400
Anthracene	0.260	0.076	0.830	1.000	0.700	0.130	0.160	0.240	0.320
Fluoranthene	2.800	1.700	17.000	15.000	12.000	2.600	3.100	4.400	5.700
Pyrene	2.000	1.100	11.000	10.000	7.400	1.800	2.100	2.700	3.800
Benzo[a] anthracene	1.200	0.500	6.400	5.000	3.300	0.910	1.000	1.400	1.800
Chrysene	2.100	1.200	10.000	8.500	5.300	1.700	1.800	2.700	3.500
Benzo[b] fluoranthene	1.600	0.940	6.000	5.400	3.700	1.300	1.300	2.000	2.600
Benzo[k] fluoranthene	0.780	0.400	3.200	2.700	1.800	0.600	0.610	0.960	1.200
Benzo[a] pyrene	1.600	0.640	6.200	5.300	3.400	1.000	1.100	1.500	2.100
Dibenzo[a.h] anthracene	1.100	0.570	4.500	3.600	2.500	0.820	0.880	1.300	1.600
Benzo[ghi] perylene	0.270	0.130	0.990	0.830	0.630	0.190	0.270	0.340	0.370
Indeno[123-cd] pyrene	2.200	1.100	8.100	6.600	4.700	1.500	1.500	2.500	3.000
**Σ 16 PAHs**	**18.010**	**9.404**	**79.385**	**73.363**	**52.973**	**13.886**	**15.523**	**22.259**	**29.162**
**Σ 7 carcinogenic PAHs**	**10.580**	**5.350**	**44.400**	**37.100**	**24.700**	**7.830**	**8.190**	**12.360**	**15.800**
c_ox _(%)	10.59	10.31	3.45	3.54	7.21	15.78	14.27	18.91	16.47

C_ox _content of organic oxidizable carbon in soil samples (%)

**Table 12:** Statistical evaluation of PAH concentrations in forest soils

PAHs	Unit	Minimum	Maximum	Arithmetic mean	Standard deviation	Geometric mean	Median	Number of samples
Naphthalene	mg/kg dm	0.100	0.360	0.210	0.100	0.180	0.190	20
Acenaphthylene	mg/kg dm	LD*	0.100	0.020	0.020	0.040	0.040	20
Acenaphthene	mg/kg dm	0.110	1.300	0.370	0.320	0.290	0.250	20
Fluorene	mg/kg dm	0.080	1.000	0.270	0.280	0.190	0.170	20
Phenanthrene	mg/kg dm	0.570	6.900	1.990	1.550	1.580	1.400	20
Anthracene	mg/kg dm	0.080	1.000	0.310	0.260	0.230	0.210	20
Fluoranthene	mg/kg dm	1.400	17.000	5.010	4.300	3.860	3.300	20
Pyrene	mg/kg dm	0.900	11.000	3.270	2.790	2.520	2.200	20
Benzo[a] anthracene	mg/kg dm	0.450	6.400	1.690	1.500	1.290	1.100	20
Chrysene	mg/kg dm	0.960	10.000	3.010	2.330	2.430	2.150	20
Benzo[b] fluoranthene	mg/kg dm	0.710	6.000	2.090	1.390	1.760	1.700	20
Benzo[k] fluoranthene	mg/kg dm	0.320	3.200	1.030	0.740	0.850	0.780	20
Benzo[a] pyrene	mg/kg dm	0.530	6.200	1.850	1.480	1.470	1.300	20
Dibenzo[a.h] anthracene	mg/kg dm	0.460	4.500	1.420	1.010	1.180	1.100	20
Benzo[ghi] perylene	mg/kg dm	0.110	0.990	0.340	0.230	0.280	0.270	20
Indeno[123-cd)pyrene	mg/kg dm	0.830	8.100	2.630	1.840	2.180	2.050	20
**Σ 16 PAHs**	mg/kg dm	7.660	79.390	25.510	19.660	20.470	18.210	20
**Σ 7 carcinogenic PAHs**	mg/kg dm	4.260	44.400	13.927	10.474	11.261	10.580	20

LD - limit of detection

Generally, high levels of the 16 investigated PAHs were identified in the transect zone of the north-south direction. The highest concentrations of the PAHs, as well as of benzo [a]pyrene and the other single PAH compounds, were detected southeast of the coal tar refinery and the town (IDs 13,14,15). The lowest concentrations were identified to the west of the town (IDs 7,12). These findings correspond to the meteorological conditions in the region, with the frequent south and north wind directions, inversion situations and the east wind direction being the least frequent.

The distribution of pollutants in the environment is influenced by landscape relief. This aspect is apparent in this region, due to the meteorological conditions low wind velocities and calm are the two most frequent wind flow situations. The altitude increases to the south and east from the town of Valasske Mezirici. In the north, the altitude decreases, and atmospheric pollutants can be better dispersed by the south wind (Figure 1). However, the PAH dispersion during frequent calm and inversion situations is very slow and is associated with pollutant concentrations increasing near the ground. These meteorological conditions are mainly correlated with low temperatures, and the PAHs are deposited on the slopes of the hills surrounding the town of Valasske Mezirici.

**Figure 8:**
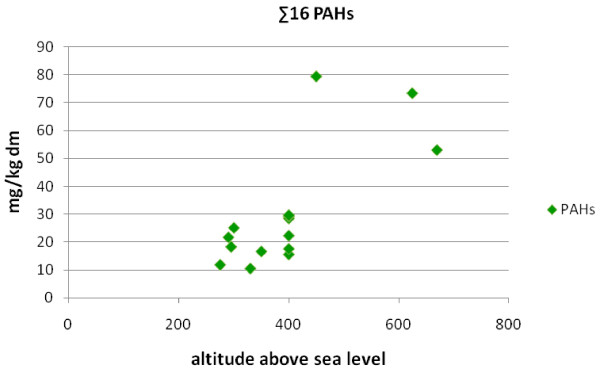
**The total PAH concentration as a function of altitude**.

The PAH concentration trend shows that the highest concentrations are obtained at higher altitudes in the proximity of the town (Figure 4 and 8). In the case of the altitude of 400 m above sea level, the PAH concentrations decrease with increased distance from the town centre; this is in agreement with other authors [39]. The three last values in Figure 8 show the PAHs and benzo [a]pyrene concentrations at the highest altitudes of 450, 625 and 670 m, to the southeast of the town (IDs 13,14, respectively 15). The site with the altitude of 450 m is the nearest to the town of these three points, which is the reason for the highest PAH value there.

For two sites at higher altitudes (500 and 550 m above sea level), the PAH and benzo [a]pyrene concentrations were not as high as in the preceding three sites. Policna (ID 7, 500 m above sea level) is located to the west of the town, outside the frequent wind flow, and the site Ruzdka (ID 16, 550 m above sea level) is the furthest from the town. It is situated to the south and is sheltered from pollutant deposition by higher hill barriers (481-691 m above sea level) between the site and the town PAH sources. The levels of PAHs and benzo [a]pyrene at the altitudes of 450, 625 and 670 m markedly exceeded other values.

Comparing the distributions of single PAHs compounds with altitude, we found that all the compounds had the same trend, except fluorene, acenaphthene and phenanthrene, where some deviations appeared. Fluorene, acenaphthene and anthracene are the least abundant in forest soil. The behavior of lower molecular PAHs is affected by the collecting effect of the bark. Due to their physico-chemical characteristics, lower molecular PAHs are more efficiently adsorbed by the bark, they are leached from the organic layers, they can be re-volatilized or biodegraded, and so on [40]. The sum of the 7 carcinogenic PAHs (according to IARC, see 3.1) in the selected forest soil ranged from 4.260-44.400 mg/kg dm, and they formed 47-61% of the sum of the 16 PAHs. The average PAH distribution pattern for forest soils is shown in Table 8 and in Figure 6. Fluoranthene, pyrene, chrysene, indeno [1,2,3-cd]pyrene and the sum of benzo [b,k]fluoranthenes are the most prominent compounds in the samples (calculated from average values) as in the urban/agricultural soil data set. The contribution of naphthalene to the sum of the 16 PAHs (0.38-1.95%) is lower than in urban/agricultural soil.

The diagnostic ratios Ind/(Ind+BghiP) and Flt/(Flt+Pyr) were also used to distinguish possible sources of high concentrations of PAHs in forest soils. The values of Ind/(Ind+BghiP), Flt/(Flt+Pyr), BaP/BghiP, Ant/(Ant+Phe) and Baa/(Baa+Chry) ranged from 0.85-0.90; 0.58-0.62; 4.07-6.39; 0.10-0.20; 0.29-0.39, respectively. These values are close to those calculated in the urban/agricultural soils in our study (Figure 7). The dominant pollution sources according to these values are pyrogenic sources, coal/wood/grass combustion, and the ratio of BaP/BghiP confirms a contribution from traffic. We can observe some differences in the Baa/(Baa+Chry) ratio, whose values are lower than 0.35, corresponding to a mixture of pyrogenic and petrogenic sources in the 10 samples of forest soils. Only pyrogenic sources are found in urban/agricultural soils using this molecular ratio. Most of the forest soil samples had a higher value of Ind/(Ind+BghiP) than the urban/agricultural soils (Figure 7).

The percentage of the sum of 8 PAH compounds (4-6) and of the sum of naphthalene and phenanthrene concentrations in the complete PAH set were in the ranges 48-63% and 5-10%, respectively. In case of the naphthalene and phenanthrene sum, we see a lower proportion in the sum of the 16 PAHs than in the case of urban/agricultural soil, whereas the proportion of the 8-PAH sum is slightly higher.

### 3.3 Comparison of the PAHs in soils from other geographic locations

The PAH concentrations in the forest soils in the Valasske Mezirici Region are much higher than the concentrations presented in [10,27,29,41]. PAH concentrations were significantly higher at upper than at lower slope sites, indicating long-distance transport [40]. The comparison of PAH concentrations in forest soils of different regions in Europe with area of Valasske Mezirici is summarized in Table 13. The PAHs concentrations presented in this paper correspond to those identified in forest soils in regions with elevated atmospheric PAH deposition: in Northern Bavaria (0.644-19.919 mg/kg dm in A-horizon) and near a blast furnace plant located in the region of Hoogovens, in the Netherlands [39,42]. The PAH levels in urban/agricultural soils are in accordance with those in industrialized areas [11,32,43-45]. In total, the PAH concentrations in forest soils were significantly greater than those identified in urban/agricultural soils.

**Table 13:** The PAHs values in various types soils in various regions

Region	Soil type	PAHs	Contents (mg/kg dm)	Literature
Norway	forest soils	Σ16 PAHs	< 006.2 - 010.0	Jensen et al., 2007,[27]
		Σ15 PAHs, except		
Austria	forest soils	Naphthalene	0.068 - 1.342	Weiss et al., 2000,[29]
UK	woodland soils	Σ15 PAHs	up to 4.850	Nam et al., 2008,[10]
Switzerland	forest soils	Σ16 PAHs	0.098 0.219	Bucheli et al., 2004,[41]
Czech republic	forest soils	Σ16 PAHs	2.000-30.000	Wilcke et al., 2007b,[40]
Northern Bavaria, Germany	forest soils	Σ16 PAHs	0.644 19.919	Krauss et al., 2000,[42]
Valasske Mezirici	forest soils	Σ16 PAHs	7.657 - 79.385	Placha et al

Belgium	industrial soil	Σ16 PAHs	2.00 - 300.000	Bakker et al., 2000,[43]
Germany	river bank soils	Σ16 PAHs	12.200 - 31.200	Pies et al., 2008,[32]
Beijing, China	urban soil	Σ16 PAHs	0.220 - 27.830	Tang et al., 2005,[44]
Nanjing, China	urban soil	Σ16 PAHs	0.290 - 17.640	Yin et al., 2008,[11]
Nanjing, China	residential areas	Σ16 PAHs	0.460 - 3.110	Yin et al., 2008,[11]
Nanjing, China	agricultural soil	Σ16 PAHs	0.310 - 27.580	Yin et al., 2008,[11]
Spain	urban soil	Σ16 PAHs	0.340 - 6.060	Nadal et al., 2007,[45]
Valasske Mezirici	urban/agricultural soil	Σ16 PAHs	0.861-10.840 (one anomalous value 35.140)	Placha et al

In their studies, Farrar et al. (2005) and Tao et al. (2007) observed clear decreasing trends in PAH concentrations in the vertical distribution in the urban boundary atmospheric layer. There are three possible scenarios of the vertical concentration profiles due to differences in emission sources, advection and vertical mixing conditions: 1) even vertical mixing, with weak fresh emissions, dominant advection and well-mixing conditions, 2) decrease with height, with ground source domination and stable atmospheric boundary layer conditions, 3) increase with height with upper boundary layer emission sources [46,47]. We can suppose, especially in the winter and autumn periods that Scenario 2 predominates in the studied area, when pyrogenic sources and traffic are the dominant pollution sources according to the determined values of molecular diagnostic ratios (see 3.1 and 3.2). Local heating and traffic are sources that emit pollutants at ground level. However, an important source of PAH pollution is located in this region the coal tar refinery. During coal tar treatment, some operations are performed (for example coal tar launching) that lead to organic compound escape to the ambient air. The escape level depends on the air temperature. The possible emissions sources in coal tar refinery are 15 m of height and they can contribute to the pollution sources at ground level not only in the winter days, but also in periods with higher temperatures.

A higher level of deposition of PAH compounds on the hill slopes in the vicinity of the town occurs in this region as a consequence of the prevailing meteorological conditions (on average, 66% of meteorological situations are calm with wind velocities 0-2.5 m/s, 27.5% have wind velocity 2.6-7.5 m/s and 6.5% have wind velocities >7.6 m/s, according to the Czech Hydrometeorological Institute) and topographical terrain. The meteorological conditions are not favorable for atmospheric pollutant dispersion, and the pollutants are accumulated in the boundary layer, the altitude of the inversion layer generally reaches a number of tens or hundreds meters) [26]. The unfavorable meteorological conditions usually persist for several days and are repeated (see 3.1).

The molecular diagnostic ratios indicate pyrogenic sources and combustion of coal, wood and grass as main pollution sources. However, the coal tar composition is formed especially from naphthalene (10% by weight), phenanthrene (4.5% by weight), fluoranthene (3.0% by weight), acenaphthylene (2.5% by weight), pyrene (2.0% by weight), fluorene (1.8% by weight), anthracene (1.3% by weight), chrysene (1.0% by weight) and acenaphthene (0.2% by weight) [48]. This fact can explain the higher concentrations of lighter PAHs, especially naphthalene, in urban/agricultural soils in the vicinity of the refinery and their higher contributions to the sum of the 16 PAHs in the urban/agricultural soils compared to the forest soils (Table 8, Figure 6). The diagnostic ratios determined for coal tar are 0.18 for Ant/(Ant+Phe), 0.58 for Flt/(Flt+Pyr), 0.54 for Baa/(Baa+Chry) and 0.53 for Ind/(Ind+BghiP) [49]. Thus, these values correspond to those in our samples in the case of Ant/(Ant+Phe) and Flt/(Flt+Pyr). The values of Baa/(Baa+Chry) were lower, and those of Ind/(Ind+BghiP) were higher in our samples. All the values correspond to coal/wood/grass combustion and pyrogenic sources. Brown et al. (2006) compared eleven samples of coal tars from 10 former manufactured gas plant sites in the Eastern United States [16]. The diagnostic ratios determined in these coal tar samples were within the ranges (Table 9): 0,22-0,27 for Ant/(Ant+Phe), 0,36-0,58 for Flt/(Flt+Pyr), 0,48-0,58 for Baa/(Baa+Chry), 0,38-0,59 for Ind/(Ind+BghiP) and 1,67-3,25 for BaP/BghiP. Thus, these values correspond to those in our samples in case of Ant/(Ant+Phe) and Flt/(Flt+Pyr). It is not possible to distinguish between pyrogenic sources and the coal tar refinery in this way, because the ratios presented are determined for cruel coal tar, but the coal tar refinery may contribute to soil by emitted PAH deposition. The diagnostic ratios in depositions can be completely different.

We observed in our samples higher concentrations of indeno [1,2,3-cd]pyrene compared to benzo [ghi]perylene. Yunker et al. (2002) listed in his work typical ratios for petroleum, single source combustion and environmental samples; the Ind/(Ind+BghiP) values were in the range of 0.09-0.70, while we determined these values to be above 0.85 in our samples, and higher in forest soils than in urban soils [49]. Indeno [1,2,3-cd]pyrene and benzo [ghi]perylene are degraded photochemically at comparable rates, and the original composition is preserved during atmospheric transport. The concentrations of benzo [a]pyrene were also significantly higher than those of benzo [ghi]perylene (4-6 times higher), and the ratio values were higher in forest soils than urban/agricultural ones. An inverse trend was observed in the case of Baa/(Baa+Chry), where mixed sources (pyrogenic and petrogenic) were determined in most of forest soils, and pyrogenic sources in the urban soils. Because we did not suppose petroleum sources in forest soils (the samples were collected in remote areas) this fact may correspond to faster benzo [a]anthracene degradation in forest soils or in the air during the atmospheric transport.

## 4. Conclusions

The urban, agricultural and forest soils in the region of Valasske Mezirici are contaminated with polycyclic aromatic hydrocarbons, which are emitted through industrial processes, transport and local heating sources. The highest PAH concentrations were found in forest soils from the forest-covered slopes of hills. The observed values were much higher than the PAH concentrations reported for forest soils in Western and Northern Europe. The PAH concentrations in the urban soils were higher than in the agricultural soils, but comparable with other soils from urban areas in the world. The highest PAH concentrations were observed in forest soils collected at higher altitudes above sea level. The PAH distribution in the mountainous region is related to altitude and influenced by meteorological conditions. The frequent calm conditions that are connected with inversion conditions contribute to PAH deposition at higher altitudes in the surroundings of the town. Elevated PAH concentrations were identified at sites further from the town, but the concentrations decreased in comparison with sites in the proximity of the town. Compared to the PAH load in other world areas, it is clearly confirmed that the soils in the region of Valasske Mezirici, especially the forest soils, are highly polluted. There is a primary source of PAH pollution in the region a coal tar refinery. There are also contributions from other sources local heating, vehicle transport and long range pollutant transport. It is not possible to distinguish the contributions of each source on the basis of diagnostic ratios.

## Abbreviations

PAHs: polycyclic aromatic hydrocarbons; Ace: Acenaphthene; Acy: Acenaphthylene; Ant: Anthracene; Baa: Benzo [a]anthracene; Bap: Benzo [a]pyrene; Bbf: Benzo [b]fluoranthene; Bkf: Benzo [k]fluoranthene; BghiP: Benzo [g.h.i]perylene; Chry Chrysene; Daa: Dibenzo [ah]anthracene, Flt: Fluoranthene; Flu: Fluorene; Ind: Indeno [1,2,3-cd]pyrene; Nap: Naphthalene; Phe: Phenanthrene; Pyr: Pyrene; Dm: dry matter.

## Competing interests

The authors declare that they have no competing interests.

## Authors' contributions

DP evaluated the results and wrote the manuscript. HR was the head of the research groupe, she designed this study and participated in preparation of the manuscript. DM prepared graphical part of the manuscript and the statistical analysis. All of three authors participated in designing of the study, in sample collecting and data evaluation. MHR participated in design of the manuscript. All authors have read and approved the final manuscript.
